# The Limits of Resilience and the Need for Resistance: Articulating the Role of Music Therapy With Young People Within a Shifting Trauma Paradigm

**DOI:** 10.3389/fpsyg.2021.600245

**Published:** 2021-01-27

**Authors:** Elly Scrine

**Affiliations:** Creative Arts and Music Therapy Research Unit, Faculty of Fine Arts and Music, The University of Melbourne, Melbourne, VIC, Australia

**Keywords:** music therapy, trauma, anti-oppressive practice, critical, adolescents

## Abstract

A broad sociocultural perspective defines trauma as the result of an event, a series of events, or a set of circumstances that is experienced as physically or emotionally harmful or life threatening, with lasting impacts on an individual’s physical, social, emotional, or spiritual wellbeing. Contexts and practices that aim to be “trauma-informed” strive to attend to the complex impacts of trauma, integrating knowledge into policies and practices, and providing a sanctuary from harm. However, there is a body of critical and decolonial scholarship that challenges the ways in which “trauma-informed” practice prioritizes individualized interventions, reinscribes colonial power relations through its conceptualizations of safety, and obscures the role of systemic injustices. Within music therapy trauma scholarship, research has thus far pointed to the affordances of music in ameliorating symptoms of trauma, bypassing unavailable cognitive processes, and working from a strengths-based orientation. In critiquing the tendency of the dominant trauma paradigm to assign vulnerability and reinforce the individual’s responsibility to develop resilience through adversity, this conceptual analysis outlines potential alternatives within music therapy. Drawing on a case example from a research project with young people in school, I elucidate the ways in which music therapy can respond to power relations as they occur within and beyond “trauma-informed” spaces. I highlight two overarching potentials for music therapy within a shifting trauma paradigm: (1) as a site in which to reframe perceived risk by fostering young people’s resistance and building their political agency and (2) in challenging the assumption of “safe spaces” and instead moving toward practices of “structuring safety.”

## Introduction

Within the behavioral sciences alone, conceptualizations of trauma have proven mutable, and the ongoing revision of diagnostic criteria for post-traumatic stress disorder has invoked controversy and debate (McNally, 2009; Pai et al., 2017; Laurel Franklin et al., 2019). The fifth and most recent edition of the American Psychiatric Association (2013) saw a substantial revision of the core diagnostic criteria for post-traumatic stress disorder (PTSD). By relocating trauma from under the anxiety disorders category and into a new standalone diagnostic category, the changes sought to remove subjectivity related to trauma exposure, limit the types of events that qualify as trauma, and provide a more objective definition of what trauma is and what it is not (Richard, 2009; Jones and Cureton, 2014; Pai et al., 2017). Modern trauma theory identifies trauma as a complex series of bodily and psychological reactions in response to danger, typically pointing to events such as sexual abuse, domestic violence, war, torture, and interpersonal violence (van der Kolk et al., 1996; Herman, 2015). Within the dominant trauma paradigm, common goals for recovery and healing include addressing threats to the sympathetic nervous system, repairing a broken or fragmented sense of self-protection, building agency and resilience, and integrating the memory of the event(s) (van der Kolk, 2014; Herman, 2015).

Less focused on the clinical utility of diagnosis, a sociocultural perspective defines trauma as the result of an event, a series of events, or a set of circumstances that one experiences as physically or emotionally harmful or life threatening, with lasting impacts on their physical, social, emotional, or spiritual wellbeing (Substance Abuse and Mental Health Services Administration, 2014, p. 7). Striving to attend more holistically to the complexity of trauma across service sectors and systems, “trauma-informed practice” is increasingly favored in community health contexts not as an intervention or treatment, but in recognition that clinical treatment alone is not sufficient (Harris and Fallot, 2001; Berger, 2019; Bransford and Cole, 2019). The Substance Abuse and Mental Health Services Administration (2014, p. 9) outlines that a trauma-informed approach (1) understands the prevalence and impacts of psychological trauma, (2) recognizes signs and symptoms, and (3) responds by integrating knowledge into policies and practices, providing sensitive care and a sanctuary from the harm. The Substance Abuse and Mental Health Services Administration (2014, p. 11) describes six core principles fundamental to a trauma-informed approach: (1) safety, (2) trustworthiness and transparency, (3) peer support, (4) collaboration and mutuality, (5) empowerment, voice, and choice, and (6) cultural, historical, and gender issues. These principles emphasize the importance of leveling power differences, moving beyond stereotypes and biases, ensuring safety, fostering resilience, and addressing historical trauma.

As I explicate in this analysis, while the principles of trauma-informed practice indicate noble intentions, they also elude to the failure of the Western trauma paradigm to account for the pervasiveness and persistence of harm embedded within our existing systems. Rather than assuming that individual clinicians working within these institutions have the capacity to ensure safety, or diffuse power differentials and deeply engrained biases, a practice that is truly trauma-informed requires a more critical analysis of power. Outlining such tensions and limitations, critical scholars and clinicians from a range of fields have sought to highlight the power relations that reside within these assumptions and noble intentions. Describing trauma as “the new colonial frontier,” Metis social worker and researcher Clark (2016a) points to the power dynamic rooted in our tendency to create a vulnerable subject who requires intervention, support, and rescue. In his proposal for an alternative approach of working with young people, African American studies professor Ginwright (2018) argues that trauma-informed care is currently “incomplete.” Ginwright outlines three limitations of trauma-informed practice: (1) its continual emphasis on trauma as an individual experience rather than a collective one; (2) its focus on treating trauma in *people* rather than considering the *social conditions* at the root of the harm; and (3) its attention to pathology and inattention to possibility (p. 2). Ginwright proposes that a shift toward “healing centered engagement” must take place, which requires practitioners to participate in “transforming the root causes of the harm within institutions” (p. 3). Informed by the work of such scholars, this conceptual analysis examines the role of music therapy in adapting to this paradigm shift. I initiate the analysis by synthesizing current discussions related to trauma in music therapy.

### Current Approaches to Trauma in Music Therapy

Research has documented the ways in which music therapy can address the relational, identity, social, and spiritual needs of clients who have experienced trauma. This body of literature has been predominantly oriented to the affordances of music in unearthing and acknowledging painful and unconscious material (Bunt and Stige, 2014; Ahonen, 2016), promoting restructuring of experiences (Krüger and Stige, 2015), adopting healthy coping behaviors (Bensimon et al., 2008), and fostering the individual’s identity outside of the trauma (Sutton, 2002). Rather than presenting a comprehensive review of the literature on the clinical outcomes associated with music therapy as a treatment for trauma, here I aim to provide a summary on the current state of practice. Commencing with two systematic reviews and one study of senior music therapists working in the trauma field, I examine how trauma is typically understood and approached as an individual health issue within clinical music therapy practice. I then introduce key music therapy frameworks that align with critical perspectives on trauma, due to their investment in social and collective action and contextualization of clients’ perceived personal problems within systemic issues.

Broadly, music therapy practice with people who have experienced trauma can be understood across several therapeutic orientations, including psychotherapeutic, cognitive and neurobiological, and resource-oriented approaches. In a recent study that explored the practices of 41 senior music therapists who work in trauma contexts, Bensimon (2020) proposed that music therapy offers a significant opportunity to attend to trauma survivors’ relational needs. The study outlined four important processes that can occur in music therapy trauma work: “musical validation,” “emotional witnessing through music,” “musical witnessing as a self-object,” and “attuned music involvement.” Bensimon’s research joins a body of scholarship that locates the benefits of music therapy as a psychotherapeutic medium for healing from trauma, cultivating the client’s sense of self, creating opportunities for externalization and internalization, and re-establishing a capacity for relatedness (Austin, 2008). Trauma-focused music and imagery is also used within the psychotherapeutic frame. Music and imagery techniques have been posited as an effective treatment for trauma symptoms, overall wellbeing, and sleep quality, and have been tested in comparison to verbal psychotherapy (Beck et al., 2018). While research into the efficacy of music as a psychotherapeutic medium often highlights the capacity for music therapists to respond to trauma as it occurs within social and cultural context, this is consistently focused on addressing the individual’s needs for social connectedness, rather than transforming the social context in which the harm occurs.

Less focused on the quality and capacity of relational needs, neurobiological approaches tend to highlight the efficacy of music therapy in controlling sensory stimulation, regulating emotions, and improving functioning (Bensimon et al., 2008). Landis-Shack et al. (2017) reviewed the use of music therapy as a treatment for trauma in adults and synthesized the fundamental cognitive, social, and neurological mechanisms that support the use of music therapy. The theoretical review opens with an explanation of the role music has played in cultivating resilience in the face of oppression, citing examples such as spiritual vocal practices of enslaved peoples in the United States of America, and community music-making in post-apartheid South Africa (p. 334). The authors note the wide range of clinical subpopulations that experience trauma, the heterogeneity of what is classified as PTSD, and the contextual factors that shape the impact of music therapy. Their examination of empirical studies builds a compelling argument for further research into music therapy as an evidence-based treatment option. However, the review by Landis-Shack et al. (2017) is ultimately focused on the potentials in music therapy for reducing PTSD symptoms, improving functioning, and as a resilience-enhancing intervention. Aligned with much of the music therapy trauma literature, the focus centers on repairing the problems that exist in the individual’s functioning, rather than examining the systems that enable a chronic lack of access to power.

McFerran et al. (2020) conducted a critical interpretive synthesis to examine the ways in which music and trauma have been connected in the research. Specifically, the authors sought to dissect the evidence for the function of music to bypass cognitive activity and stimulate more primitive neural regions. Outlining the ways brain-based explanations appear to neatly solve the complex terrain of trauma, McFerran et al. (2020, p. 6) point to a “reductive trend” in approaches that position trauma only as a malfunctioning of the individual’s cognition and psychology. The review categorizes the use of music-based methods into four different purposes: stabilizing, entrainment, expressive, and performative. Calling for greater attention to reflexivity in trauma research, McFerran et al. (2020) emphasize the value in approaches that do not treat music as an objective variable, but rather a context-bound resource that affords possibilities and can enact social critique. In summarizing potential future directions, the authors point to the resources that lie in critical theory and scholarship outside of the trauma field which emphasize the social conditions that enable trauma, rather than focusing only on the individual’s adverse life experiences.

### Relevant Music Therapy Frameworks

A primary goal of modern trauma-informed practice lies in building clients’ agency and resources, an aim that is congruent with a range of music therapy approaches and orientations. The ways in which community music experiences can foster and cultivate individual and collective agency has been explored in a dedicated field of music therapy and musicology scholarship (Ramsey, 2003; DeNora, 2004; Kenny, 2006; O’Grady et al., 2014; Bain, Grzanka and Crowe, 2016; Zarate, 2016). There are two broad music therapy frameworks specifically relevant to critical perspectives on trauma, which divest from pathology and deficit discourses and emphasize the affordances of collective action. Community Music Therapy (Pavlicevic and Ansdell, 2004; Stige and Aarø, 2012) practice and research has brought the role of music therapists into the social domain, favoring social action over individualized interventions and clinical goals.

A participatory action pilot study by Thomas (2020) with adolescents from limited resource communities is an example of Community Music Therapy in both research and practice. In the study, Thomas reframes Black/African American young people’s trauma and associated behaviors in the context of systemic racism and socioeconomic oppression. The research sought to understand the role of community musicking in the young people’s lives and involved the participants in each aspect of decision making. The goals of the sessions were continually set and revisited in collaboration with the young people, and the practices prioritized the young people’s own music cultures and preferences. Thomas (2020, p. 117) underscores the importance of clinical work moving toward community-based approaches that are client-led and that position the individual/group within a holistic continuum. Also seeking to ground clients’ participation and experience in social context, Resource-Oriented Music Therapy (Rolvsjord, 2010) built on the tenets of Community Music Therapy, journeying beyond entrenched patient/expert power dynamics, and focusing on the *quality* of clients’ participation and experience. Both of these music therapy frameworks critique the pathologization and “treatment” of individuals, and advocate for a contextual model that includes “relational, structural, and community levels in our conceptualizations of therapy” (Rolvsjord and Stige, 2015, p. 59). Both frameworks adopt a critical stance that contextualizes music therapy as it occurs within complex systems and socio-political conditions (Edwards, 2011; Ansdell, 2014), and views clients as active agents of change, rather than passive recipients of “help” (Rolvsjord, 2015).

This music therapy scholarship actively challenges the oversimplification of young people into categories of risk and deficit (Thomas, 2020), promotes collaboration (Bolger, 2015), and seeks to actively decenter narratives of victimhood in favor of young people’s resources and resilience (Pasiali, 2011; Fairchild and McFerran, 2018). Researchers have demonstrated the unique role that music therapy can play with communities of young people who are marginalized along axes of race and class (Hadley and Yancy, 2012; Leonard, 2020; Thomas, 2020), and as an affirmative, political space for queer and gender diverse young people (Bain et al., 2016). Within this scholarship, there are calls to action to be mindful of the language and discourse surrounding clients’ perceived vulnerabilities and risk (Fairchild and Bibb, 2016) and to strengthen our focus on the social conditions that construct and reinforce pathology (Baines, 2013). In an example of such, research by Norris (2019) explored the aesthetic experiences of Black music therapy clients with chronic pain, using critical race theory to articulate the historical, structural, and cultural context of chronic pain and health care disparities. Norris outlines how, in an effort to legitimize and strengthen an evidence-base, the music therapy field has prioritized medical and outcome-based research which inherently privileges white narratives and centers Eurocentric ideologies. In examining anxiety not as a clinical symptom but as a larger relational experience, Zarate (2016) urges music therapists to begin to reconceptualize anxiety as it occurs in a context of competitive individualism and unjust power structures. Zarate (2016, p. 5) writes, “…simply attempting to soothe anxiety as a one-dimensional operating symptom that is not in relationship to someone or something is a misrepresentation of the complex causes and impacts of anxiety.” Zarate identifies music therapy as relational, multisensory process that holds immense potential as an approach for working with anxiety from a social and cultural perspective. While there have been calls for music therapy scholarship to attend to the contexts and circumstances that create and perpetuate trauma (Fairchild and Hadley, 2018; Hadley, 2020; McFerran et al., 2020; Thomas, 2020), thus far, this has not been a prominent focus in the music therapy trauma research.

## Conceptualizing the Argument

Although trauma-informed practice aims to engage with the individual’s context and address power differentials between client and therapist (Substance Abuse and Mental Health Services Administration, 2014, p. 11), dominant contemporary trauma frameworks rarely examine the social conditions that underly clients’ perceived “risk factors.” Perspectives that highlight the coloniality of the dominant trauma paradigm are particularly relevant here because they illuminate the power relations that reside within the seemingly benevolent. Efforts to challenge the Eurocentrism inherent in the trauma field seek to shift the focus away from problems in people’s minds, and into injustices in laws, policy, and governance that result in structural violence. These arguments have been explicated by scholars in settler colonies such as Australia (Atkinson, 2002), Aotearoa/New Zealand (Pihama et al., 2014), Canada (Linklater, 2014; Clark, 2016a), and the United States (Gone, 2013; Menakem, 2017). While situated across distinct contexts, engaging with these researchers’ work reveals some common themes regarding the dynamics of erasure in the trauma paradigm and mental health professions. Most often, these scholars stress that the prevalence of trauma in Indigenous communities should be more critically understood within a context of colonization, conquest, and dispossession. Further, they underline how these are more than legacies of subjugation to reflect on historically, as colonization is a structure rather than an event (Wolfe, 2006). This understanding prompts us to attend not only to the colonial power relations that are deeply embedded in the institutions, structures, laws, and policies of all settler nations but also in our contemporary everyday actions and interactions.

Such perspectives incite a profound question as to how practice that endeavors to be “trauma-informed” can ethically do so without deep and ongoing engagement with power relations such as racialization and colonization. This is especially pressing given the wealth of data that details the disproportionate level of trauma, as it is generally defined, experienced by Indigenous peoples and racialized communities globally (Kukutai and Taylor, 2016). Paradies (2016, p. 88) has operationalized colonization as a driver of Indigenous ill-health and summarized the impacts of the “colonial mentality” as a form of internalized racism that is associated with low self-esteem, anxiety, and depression. The Kirmayer et al. (2014) model was pivotal here in conceptualizing the ongoing and interrelated impacts of historical trauma. The model demonstrates the relationship between political disempowerment (genocide and dispossession of land), with community loss (dysfunction, conflict, and stereotyping), familial issues (loss of children, grief, domestic violence, and abuse), and individual impacts (denigration of identity, suppression of culture, mental health issues, and difficulty parenting). The American Academy of Pediatrics has declared racism a core determinant of child health, and linked its impacts to birth disparities, chronic health conditions, and mental health issues in children and young people (Trent et al., 2019). Despite the ubiquity of statistics in education, health, and mental health contexts that point to the “vulnerability” of these communities, the construction and maintenance of what is “trauma-informed” continues to be dominated by white, Western perspectives. While trauma is increasingly at the center of literature and practice from social work, psychology, psychiatry, other therapeutic disciplines, and educational contexts, it is still rarely acknowledged that its definitions and theoretical frameworks are rooted in a raced, classed, abled, gendered hegemony.

Clark (2016a, p. 2) highlights how in clinical and therapeutic contexts, the continual identification of marginalized people as “traumatized” has constructed a colonial power relation, in which there is a subject who requires intervention, support, and saving. In the school context, critical youth studies researcher Emdin (2016) has also pointed to a parallel dynamic that occurs between well-meaning educators with Indigenous young people and students of color. While simultaneously demonstrating their care for “at risk” students (who are predominantly students of color), professionals (who are predominantly white) downplay young people’s assets, multiplicity, and own cultural resources, and position themselves as “protectors” of the young people’s wellbeing. Clark points out how the statistics that invoke shock and horror associated with Indigenous children and young people’s trauma only strengthen these kinds of dynamics, justifying ongoing intervention and control. In the Australian context, Palawa scholar Walter (2016) points to the illusion of neutrality in numbers that are frequently wielded against Aboriginal and Torres Strait Islander communities, creating an intensively pervasive and readily available deficit discourse. Scholars such as Roestenburg (2010), Kirmayer et al. (2014), and Hogarth (2017), each situated across different settler-colonies, have articulated some parallels in how deficit discourse functions. In continually referring to Indigenous peoples only in relation to risk and vulnerability, and deflecting attention from ongoing material dispossession, the risk and vulnerability associated with these communities becomes naturalized, while structural factors are obscured. Through this lens, “vulnerable” individuals’ experiences of trauma can thus be understood as an unfortunate but natural consequence of the risk factors associated with their community. In the Australian context, Goenpul scholar Moreton-Robinson (2009) outlines the role that pathologization plays in these dynamics, where a white sovereign right to discipline “dysfunctional” Indigenous people are exercised in the name of virtue. Informed by Moreton-Robinson’s argument, Gilbey and McCormack (2018) articulated this “trick” of pathologization in relation to trauma:

“Through sleight of hand, like a master magician, they say, “Look over here,” as the deception happens while your eyes are averted. This is exactly what they were doing by “pathologising” and problematizing individual communities and people. This is the cloak of invisibility, because almost anything can be justified in the name of benevolence (p. 138).”

## Positioning This Analysis

Critiques of the dominant trauma paradigm have been led in particular by decolonial and critical race scholars and clinicians, and I center this body of literature for several reasons. In an effort to challenge the Eurocentric hegemony of trauma research, I privilege research conducted by First Nation scholars, Black scholars, and scholars of color, on issues impacting their own communities. As critical race theory has demonstrated, racism is not simply interpersonal prejudice or discrimination but occurs institutionally and systemically (Yancy, 2016; Delgado and Stefancic, 2017). Overt and covert forces of white supremacy and colonialism produce violence that occurs individually, collectively, and structurally, and is compounded by other forms of violence including ableism, classism, heteronormativity, patriarchy, and cissexism. These forces are rarely discussed in the dominant trauma literature. There have been calls to bring an explicit light to the presence of colonial structures within trauma practice, as well as in health care, research, and pedagogy more broadly. Public health experts in the Lancet medical journal have proposed that a focus on structural racism offers a material and achievable approach to increasing overall health equity and improving population health (Bailey et al., 2017). The impact of structural racism on health and education outcomes has been demonstrated not only in public health research (Paradies, 2016) but also in recent social and political uprisings against racial injustice taking place globally (Cave et al., 2020). Gipson et al. (2020) write, “Racialized violence and multi-contextual disparities are not new; however, they are swelled and this swelling has led to the current eruption of uprising.” Several music therapy scholars have emphasized that without a conscious and active view and understanding of ourselves as racialized people within historical, sociocultural, pedagogical, and academic contexts that have centered whiteness, we will perpetrate racism (Norris, 2019; Leonard, 2020; Thomas, 2020). Such a position calls into question whether we can simply move “past cultural stereotypes and biases” from within trauma-informed practice (Substance Abuse and Mental Health Services Administration, 2014, p. 11). As Thomas (2020) has highlighted, doing so requires us to examine the persistence of violence and harm that occurs in ways that are systemic, complex, and that we may be complicit in.

As a white, queer academic and clinician located on unceded Indigenous land in Australia, it is necessary to position myself as a beneficiary of structural power, and to activate my responsibility to understand and challenge how academic and health care institutions function in relation to colonial dynamics. While crucially informed by decolonial scholarship, my aim is not to “decolonize” trauma-informed care, or music therapy. I wish to make this point explicit, because this conceptual analysis comes at a point of palpable academic and institutional attention toward “decolonization” (Doharty et al., 2020). Within this context, I am mindful that efforts and agendas to “decolonize” that are assumed by white and non-Indigenous individuals, institutions, movements, and professions tend to be engaged with superficially. Often, these efforts are led in ways that not only attempt to reconcile settler guilt but also reinforce existing power dynamics (Tuck and Yang, 2012). White scholars have much to gain from adopting the language of decolonization, with very little accountability for adopting anything but the language (Dar et al., 2018).

The overuse, appropriation, and commodification of “decolonizing” discourse do not render decolonization any less urgent. Rather, it necessitates careful delineation of the difference between superficial engagement vs. genuine disruption and overturning of power structures (Doharty et al., 2020). Further, it requires a deep and genuine examination of whiteness and the practices of scholars who produce knowledge in ways that uphold white structures. As I outline below, existing critiques emphasize a need to better account for and respond to violence as it occurs in ways that are normalized, covert, or rendered inconsequential. By drawing together key arguments that challenge dominant, Eurocentric responses to trauma, I do not claim my arguments below as original, nor emerging. Rather, informed by these existing arguments, I seek to shift the trauma discourse in music therapy to focus on the conditions that enable trauma. I propose that in developing discussions informed by critical perspectives, music therapy not only has the potential to better understand and respond to trauma but also a unique role to play in shifting the current trauma paradigm.

## Trauma Under Neoliberalism and the Problem with “Resilience”

Trauma is ultimately understood within the dominant paradigm as an individual health problem. Vulnerability discourses can also be understood as a function of neoliberalism, concealing the social conditions in which trauma occurs, and placing responsibility on the individual. Because neoliberalism redirects social responsibility into individual responsibility, disadvantage can be converted into a consequence of weakness, personal failure, or incompetence. In designating vulnerability onto particular identity groups, structural violence can be transformed into personal responsibility through the use of compassion and care (McLaughlin, 2012). This invokes questions for those of us in the “helping” professions because it is our care and compassion for the most “at risk” groups that is mobilized. By vulnerabilizing people and the harm they have experienced, we focus only on the suffering of the individuals, rather than the systems that perpetuate harm. As an example, critical race scholars have pointed to a particularly problematic racialized dynamic in institutional contexts, where young Indigenous, Black, and people of color are expected to disclose their tales of harm and victimhood to white professionals, who consider themselves individually capable of “saving” these young people (Razack, 1998; Emdin, 2016).

Switching focus from client populations and onto the clinical and community workers ourselves, the notion of “vicarious trauma” is a relevant example to point to here. Significant attention has been paid to the transmission of trauma, vicarious traumatic stress, burnout, and compassion fatigue among therapists and community workers (Clements-Cortes, 2013; Fairchild, 2018; Downs, 2019). The literature even highlights how the creative arts therapies can be a resource for health workers in the face of burnout (Kacem et al., 2020; Reed et al., 2020). Again, however, I point to the ways in which these can be understood as individualized rather than collective struggles. For example, burnout in the trauma context is often conceptualized as arising from a therapist facing the limits of how much of their clients’ trauma they can hold and care for, before it begins to impact their own wellbeing. I am not querying the prevalence of exhaustion, frustration, and harm that health and community workers experience. Instead, I propose a broadening of the spotlight to reveal more than only the individual. I draw on Reynolds’ (2011) critique of burnout discourse, which emphasizes that the source of burnout is not the trauma that we perceive to reside *in* clients. Reynolds encourages therapists to look instead to the *contexts of injustice* in which our clients exist, and the conditions we work within that fail to protect people from harm. This expansion aligns with Ginwright (2018, p. 2), who articulates the limits of only focusing on the harm experienced by the individual, whereby “we only address half of the equation leaving the toxic systems, policies, and practices neatly intact.” In Ginwright’s “healing centered engagement” model, the adult professionals who attempt to heal and support young people are also supported through their workplace policies and organizational structures. Understanding burnout and potential trauma-relation dynamics from this particular widened context supports the therapists’ understanding of the therapeutic processes. Further, it develops our understanding of the dynamics between the therapist-client, the impacts of systemic structures on our health and wellbeing, and what is required for practice sustainability.

When responses to trauma locate harm only in individual psychology and physiology, resilience and “grit” discourse can be understood as yet another means of individualizing societal and systemic problems. Increasingly, across clinical and community contexts that aim to be trauma-informed, psychological concepts are used to justify the need for building young people’s resilience (Pollard, 2014). Rather than advocating for material change or transformation, young people who are marginalized by conditions of social inequality, such as racism, poverty, and violence, are expected to learn to adapt (Shaw et al., 2016). As such, the development of resilience is identified as a trait or competency that young people most impacted by systemic oppression are expected to develop (Brunila and Rossi, 2018). The rise of what Brunila (2012) terms “therapization” in education means that young people who are identified as “at risk” are provided with targeted interventions that seek to build their resilience in response to trauma. In what McLeod (2012) deems “psychologization,” psychology is provided as the answer to problems of social conditions. Summarizing the failure of the trauma paradigm to fully capture the impacts of historical trauma, Kirmayer et al. (2014) note how individual therapy is framed as the answer for collective healing. Within resilience culture, “grit” is encouraged and celebrated, emphasizing the individual’s hard work, adaptation, and success in spite of adversity. Saltman (2014) articulates the ways in which grit functions with young people in education contexts:

““Grit” is a pedagogy of control that is predicated upon a promise made to poor children that if they learn the tools of self-control and learn to endure drudgery, they can compete with rich children for scarce economic resources. Proponents of teaching “grit” contend that the poor are biologically and psychologically traumatized by poverty. The trauma of poverty, they argue, can be overcome through learned self-control and submission to authority within the school (p. 43).”

Through a reliance on resilience and grit as an antidote to trauma, the conditions under which harm occurs are obscured, and instead, children and young people are taught to adapt with tenacity and resourcefulness. In the education context, music has been identified as a potential site of resilience-building for “at risk” youth (Barrett and Bond, 2015), though Hess (2019) notes that music can both disrupt and reinforce a discourse of vulnerability. Songwriting and hip hop pedagogy have been used to actively challenge these discourses within music therapy (Thomas, 2020), and have been posed within music education contexts as a form of “counterstorytelling” (Pulido, 2009; Hess, 2018, 2019; Steele, 2020). This signifies an important departure point for this conceptual analysis. Seeking to move beyond promoting young people’s resilience, this dedicated field of critical education scholarship instead articulates the affordances of songwriting in fostering young people’s political agency and collective action (Hess, 2019), negotiating and challenging the ways in which they are racialized (Pulido, 2009; Steele, 2020), and as a culturally-affirming site for cultivating mental health (Emdin and Adjapong, 2018).

## Discussion

Music therapy offers transformative potential as a relational, embodied, and multidimensional sensory experience that can be deeply grounded in context (Rolvsjord and Stige, 2015) and culturally responsive (Norris, 2019; Moonga, 2020). In the remainder of this conceptual analysis, I outline an example of music therapy as a site in which to attend to the social conditions and power dynamics that enable and maintain trauma. The case example draws on a critical ethnography research project that I conducted to examine the role of group music therapy in exploring gender and power with young people in school (Scrine, 2018, 2019; Scrine and McFerran, 2018). In this research, the vast majority of the participants were considered to be “at risk,” and the subject of trauma arose regularly throughout the program itself, as well as in the ways staff described the young people during interviews. I will focus particularly on two of these groups in order to highlight two overarching potentials in music therapy: (1) in reframing perceived risk as a site for resistance and agency-building and (2) in constructing a space in which safety is not assumed, but consciously and continuously structured. I commence with a description of the context and the ways trauma was understood in the setting.

### Case Example: South East College

The site of the research was South East College,^1^ a coeducational and multicultural government school located in a low socioeconomic area in the outer urban suburbs of Melbourne, Australia. During the research project setup, staff proposed that music could an effective way to build resilience within in the student community where there was high incidence of family violence, substance abuse, intergenerational poverty and unemployment, and intervention from Child Protection (Scrine and McFerran, 2018, p. 56). Interviews with welfare and teaching staff established that they did not believe that the students’ challenging behavior at school was a reflection of their inherent hopelessness, disobedience, or disengagement. Rather, it was understood to be a consequence of the trauma they experienced at home (Scrine and McFerran, 2018, p. 54). Alternatively, themes that emerged from interviews constructed the school as a place of care, neutrality, and safety (p. 78). Such narratives aligned with colonial ideals of institutional benevolence, where schools are seen to give young people “a better life,” and individual staff are seen to be capable of saving them (Emdin, 2016, p. 20).

One group consisted of 13-year-old boys^2^ who were referred to music therapy due to issues with violent behavior at school. The welfare team at South East College initially posed whether the music therapy program could focus on socioemotional goals for the boys’ “anger management” (Scrine, 2018, p. 101). Another group consisted of 14-year-old girls who were referred due to ongoing conflict between them, and the welfare team anticipated the music therapy program would function as a site to develop their relational skills and support a culture of respect and kindness between the girls (p. 92). Throughout the initial interviews with staff, there was a significant focus on the young people’s socio-cultural communities, experiences of poverty, and families as “risk factors.” Within this context, I identified a need to challenge the construction of music as a sanctuary for the young people to build resilience in the face of these perceived “risks”, or a psychoeducational opportunity to develop social/emotional competencies. Instead, I positioned music therapy as an opportunity to foster the young people’s own political agency.

### Beyond Risk and Into Resistance

A common thread running through critical and decolonial trauma scholarship is the importance of highlighting agency and resistance over victimhood, and of connecting personal struggles to structural issues. In the case study context where the students’ social, emotional, and behavioral issues at school were understood to be a result of trauma and dysfunction, the music therapy group program did not focus on emotional regulation, nor an expectation that the young people should share their experiences of trauma in the group. Rather, informed by existing critical music therapy frameworks (Rolvsjord, 2010; Stige and Aarø, 2012; Baines, 2013), the group goals centered on building the young people’s agency, exploring power differentials, and repositioning their perceived vulnerabilities in relation to broader systemic forces (Scrine, 2019).

For the girls group whose members were involved in conflict regarding each other’s sexuality and relationships with their male peers at the school, this meant establishing a dynamic of solidarity between them. Throughout the program, we used music as a way to explore their relationships with each other in the context of gender and power. This approach was informed in particular by the body of work led by critical feminist scholars and youth practitioners who highlight the emancipatory potential in girls’ groups (for example, Brown, 2009; Clark, 2016b; Stanger, 2016; Renold, 2017; Showunmi, 2017). In the boys group, we explored expectations related to masculinity and emotion, as well as the deficit discourses they experienced in relation to race, class, and disability. Both groups drew on music therapy techniques including songwriting and improvisation to afford opportunities for the young people to connect their individual experiences to the structures and institutions that impacted their lives. During interviews, one of the participants described this as “actually talking about what happens in the real world” (Scrine and McFerran, 2018, p. 59). This was not an evasion of the trauma and violence the young people may have experienced in their homes, but rather an expansion to attend to the trauma of structural violence. Clark (2016b) articulates this shift from a *trauma*-informed approach to a *violence*-informed approach:

“A violence-informed and intersectional girls’ group locates the source of girls’ challenges within structural and systemic problems such as racism, poverty, sexism, and the intersections of these in their lives. We support the young women in healthy resistance to these problems, and in their efforts to move back into connection with themselves and others. We do this through a range of violence-informed strategies of naming, educating, and supporting healthy resistance strategies (p. 11).”

Interviews with the young people sought to articulate the way they understood the role of music therapy when adopting such an approach, which revealed three interrelated themes (Scrine and McFerran, 2018). First, the young people described relishing a space where their ideas about the broader structural forces that shaped their individual experiences were valued (p. 58). Second, the participants identified the music therapist’s role as unique in regard to the way power can be shared rather than weaponized (p. 60). Third, the young people pointed to the ways in which this approach had both emancipatory potential and complex consequences in the context of neoliberal schooling institutions (p. 61).

### Beyond “Safe Spaces” and Into Structuring Safety

Notions of safety are often at the forefront of trauma literature. In this section, I draw particularly on the work of decolonial scholars and clinicians such as Clark (2016a, 2017) and Richardson and Reynolds (2014), who have urged clinicians to reconceptualize “safe spaces” in trauma work. Clark (2017) proposes that the way we understand “safety” in therapeutic and community spaces actually reinscribes power relations. Clark argues that for many clients in therapeutic spaces, dominant understandings of safety are irrelevant and harmful. In relying on the notion that clients can actually experience safety from violence, we reinscribe a Western perspective that situates trauma as a single, catastrophic event. In the music education context, Hess (2019, p. 7) eludes to “safe spaces” as all but a fantasy, especially given that most education and arts programs fail to consider *who* dictates safety, and *for whom* spaces are safe. For those who experience violence under policies and systems, and for whom trauma is collective, historical, and ongoing, critical trauma scholars propose new ways of understanding “relative safety.” This requires developing what Richardson and Reynolds (2014) describe as a practice of “structuring safety.”

Music therapists’ expertise exists at an intersection of clinical, relational, and musical skills that can be drawn upon when attempting to structure safety. One of the key distinctions that separates music therapy and other creative arts therapies from verbal therapies is that individuals shift from “subject” or “object” and into “active participant” (Ansdell, 2003; Rolvsjord, 2015; Norris, 2019). During interviews in the case example research, the participants described the benefits of music therapy in the context of trauma: “Kids might have trouble, saying what actually happens. So they might need to express it in a musical form” (Scrine and McFerran, 2018, p. 59). In addition to verbal counseling skills and an orientation that fosters clients’ strengths and resources, music therapists harness tangible elements of music such as pulse, tempo, and rhythm that can be continuously modified and shaped to provide structure and predictability. The systematic review by McFerran et al. (2020) provides the most relevant synthesis here, as the notion of safety appears in each of their four categories of approaches to music and trauma. Articles collected into the stabilizing category point to the mechanisms of regulation and the way prosodic stimulation can signal safety (p. 9). These theories are deeply intertwined with entrainment and synchronization theory. Many theoretical and practice disciplines explore and are informed by entrainment and the ways music can stabilize and synchronize auditory, motor, and emotional domains of human function. Indeed, in the trauma field, rhythmic stimulation is used as a form of regulation, both from a somatic psychotherapeutic perspective to make contact with dissociated experiences (Ogden and Fisher, 2015), and as a sensorimotor grounding tool to bypass unavailable cognitive processes (Hasler, 2017). However, music therapists can structure safety not only in a mechanical, functional, or behavioral sense by influencing the autonomic nervous system, but also by challenging power dynamics and ways the in which harm is replicated in therapeutic spaces.

If trauma is understood as a loss or devaluation of individual, cultural, or collective agency, structuring safety in therapeutic work requires developing a culture of consent between therapists and clients. Richardson and Reynolds (2014) propose that doing so requires a fundamental shift away from the assumption that professionals are entitled to hearing their clients’ stories, or that it is a client’s responsibility to bare their story in order to truly benefit from therapy. Fostering agency requires a continual navigation of consent, opportunities for clients to exercise choice and control, and for those choices to be validated and affirmed. When navigating disclosures of harm within a practice of structuring safety, Richardson and Reynolds urge therapists to strive for curiosity about the survivor’s acts of resistance against their suffering and oppression, rather than only seeking to evoke stories of their pain and victimization (p. 157). During one session of the girls group in the case example research, several of the participants disclosed experiences of sexual assault, and expressed that this form of violence was normalized in the school. A focus for the group then became documenting their resistance strategies, not only within their experiences of interpersonal violence, but within a school context and broader culture where such complaints are often dismissed or trivialized. As Clark (2017) emphasizes, acts of resistance should be witnessed and celebrated not only when they occur in contexts of interpersonal harm or abuse, but also when they oppose institutional forces. These practices actively challenge neoliberal forces that detach the individual from their context, obscure the role of systemic factors, and create identity-based “vulnerabilities.” Instead, they highlight the importance of recognizing the client’s whole self and broader community. Rather than assuming that any individual therapist has the capacity to create a space that is automatically safe, this shift prioritizes the resources brought by the client, locates the strengths of their community, and structures safety through consent, collaboration, and choice.

Notions of choice and control are central in music therapy, where the elements of music provide a framework within which choices can be made continuously, and contributions can be written, accentuated, repeated, re-mixed, and experimented with under the client’s direction. Because music therapy often involves the client participating in some form of creation, whether this is a playlist, an improvisation, or an original or existing song, there are repeating and ongoing opportunities for choice. Music therapists’ expertise lies in assessing where and how choices can be offered and highlighted, and scaffolding music-based experiences that are attuned to these needs. For example, a music therapist can recognize when a client may benefit from the opportunity to simply make a choice about which instrument to play during an improvisation, or which song to sing together. Or, they may facilitate an entire songwriting and recording experience which offers abundant opportunities for choice, structure, and externalization. During improvisation, a therapist can highlight the client’s musical motif by playing it back with greater volume or at a slower tempo, or provide a steady, predictable accompaniment for the client to improvise over and return to. During interviews in the research project, one of the participants in the girls group summarized the concept of safety in music therapy:

“It didn’t feel like we had to, but you were like, asking. So if we wanted to tell you, we did tell you. And I think it was because we were together, and you asked us questions and we were talking and stuff… because normal space, normal classroom, you just walk past each other. But we was just *doing* it, just there. And we were writing stuff down, and making music and stuff” (Scrine and McFerran, 2018, p. 61).

## Conclusion

There is a range of scholarship that points to the value of music in a shifting trauma paradigm. Trauma research in music therapy has predominantly focused on ameliorating symptoms, working from a strengths-based orientation, and the relational, embodied, and multisensory potentials of music. This literature has emphasized the potentials of music in bypassing cognitive activity to stimulate primary structures within the limbic system, as well as in fostering relational competencies, attunement, and healthy parts of the self. However, there have been recent prompts for music therapists to consider the social conditions that enable trauma, and a dedicated body of research encouraging all practitioners to attend to the power relations inherent in the ways we identify particular communities of young people as “traumatized” or “at risk.” As critical and decolonial trauma scholars have noted, the dominant trauma paradigm has the potential to further perpetuate harm, and does so through assigning vulnerability, reinscribing colonial power dynamics, and reinforcing individual responsibility. These perspectives tie together a body of work that challenges “trauma-informed” practices that prioritize individualized clinical interventions, assume that safety can be guaranteed, and obscure the role of systemic injustices. Within music therapy, there are pre-existing frameworks that seek to locate people in context and connect personal struggles to broader systemic and institutional forces, though thus far, these have not been the focus of trauma research. Drawing on case study research, I have outlined two overarching potentials for music therapy within a shifting trauma paradigm.

First, music therapists can actively challenge discourses of risk and instead foster young people’s resistance and collective consciousness-building. In contexts where young people are positioned as vulnerable, dysfunctional, and in need of intervention and regulation, a “violence-informed” approach locates the source of the young people’s challenges within the intersections of a range of systemic issues (Clark, 2016b). Music therapy practices such as group songwriting can function to name these broader forces, document healthy resistance strategies, and reposition young people as political agents. An example of this repositioning in the case study provided was the “girls group.” Initially posed as a psychoeducational opportunity for the young people to develop their relational skills, the girls group instead was a site in which they explored their conflict and experiences of violence in the context of broader cultures of misogyny and sexual violence.

The second overarching potential for music therapy within trauma work with young people lies in moving away from the assumption of safe spaces, and into practices of structuring safety. The young people in the research described the capacity of a music therapist working from a violence-informed approach to share power and hold space, and they saw music therapy as a site in which their reflections and responses to the structural inequalities that shaped their lives were taken seriously. This is a journey away from power relations that position individual professionals as entitled to young people’s trauma stories (Richardson and Reynolds, 2014) or capable of “saving” them (Emdin, 2016). In structuring safety, practitioners continuously navigate consent, create opportunities for clients to exercise choice and control, and remain curious about their acts of resistance in the face of oppression. Music therapy offers powerful potential here as individuals move from subject or object and into an active participant. At music therapists’ disposal lies not only the elements of music that afford structure, predictability, and flexibility, but also infinite opportunities to offer choice and control, which are crucial in cultivating a culture of consent between therapist and client.

The benefits of music for those impacted by trauma can extend beyond “building resilience.” This is significant, because when prioritized as a competency in settings that lack a willingness or capacity to name forces of systemic oppression, “resilience” can function to serve neoliberal ideologies and a pedagogy of control. By locating trauma only in people’s minds and not in the institutions, policies, and systems that create conditions for harm, responsibility is placed on the individual to adapt and survive. Where music and the arts in general are assumed to be safe havens and sanctuaries detached from trauma and harm, we fail to recognize the power dynamics inherent in these spaces. Music can and should be understood as a tool through which to actively name and respond to these dynamics. When harnessing the affordances of music, while responding to power relations as they occur within and beyond therapeutic spaces, music therapists have a powerful role to play in shaping the future of trauma work that is more critically informed and truly responsive.

## Ethics Statement

The studies involving human participants were reviewed and approved by Melbourne University Human Research Ethics Committee (HREC) and the Victorian Department of Education. Written informed consent to participate in this study was provided by the participants’ legal guardian/next of kin.

## Author Contributions

The author confirms being the sole contributor of this work and has approved it for publication.

### Conflict of Interest

The author declares that the research was conducted in the absence of any commercial or financial relationships that could be construed as a potential conflict of interest.

## Footnotes

^1^This is a pseudonym

^2^The gendered descriptors reflect the terms requested by the participants at the time of the research
